# A High-Fat and High-Carbohydrate Diet Promotes Reminiscent Hallmarks of an Aging Ovary in the Rabbit Model

**DOI:** 10.3390/biomedicines10123068

**Published:** 2022-11-29

**Authors:** Verónica Díaz-Hernández, Luis M. Montaño, Ivette Caldelas, Alejandro Marmolejo-Valencia

**Affiliations:** 1 Departamento de Embriología, Facultad de Medicina, Universidad Nacional Autónoma de México, Mexico City 04510, Mexico; 2 Departamento de Farmacología, Facultad de Medicina, Universidad Nacional Autónoma de México, Mexico City 04510, Mexico; 3 Departamento de Biología Celular y Fisiología, Instituto de Investigaciones Biomédicas, Universidad Nacional Autónoma de México, Mexico City 04510, Mexico

**Keywords:** ovarian aging, hypercaloric diets, follicular decreased, oxidative stress, NF-ΚΒ, interstitial gland, senescence

## Abstract

The primary definition of ovarian aging refers to the loss of follicles. Moreover, the aging of the microenvironment in ovaries, specifically affecting the follicles, may reveal deterioration with advancing age. Besides aging, metabolic disorders associated with hypercaloric diets may affect ovarian health and manifest characteristics associated with premature aging. In this study, we used 10-week-old chinchilla rabbits fed with a high-fat and high-carbohydrate diet (HFCD) until 25 weeks of age to explore hallmarks of reminiscent ovarian aging. The HFCD diet appeared to affect the ovarian reserve, reflected in a significant decrease in primordial follicles. Likewise, Sudan black stain detection revealed substantial differences in the deposits of lipofuscin in the interstitial glands of HFCD-fed rabbits compared to controls, constituting a “hallmark” of aging. The HFCD showed no induced changes in the expression of SOD 2 in the interstitial gland; however, surface epithelium cells were greater expressed. Besides this, the HFCD induced nuclear translocation of NF-ΚΒ p65 factor transcription in surface epithelium cells. We conclude that an HFCD induces a greater accumulation of senescence cells in the interstitial gland, promoting characteristics reminiscent of ovarian aging. However, the activation mechanism of NF-KB caused by an HFCD, which may be stress-responsive and generated by the interstitial gland, requires further study.

## 1. Introduction

The aging ovary is an exceptional case compared to the other organs in the body when undergoing aging. The ovary is not functional in all lifespans and is not indispensable for vital function. However, recent studies have revealed the connections between ovarian aging and systemic aging, so during menopause, signs of structural and functional alterations in women initiate [1].

The ovary controls female fecundity, as it is responsible for growth, follicular maturation, and the hormonal sexual cycle. Ovarian aging entails a decline in the quantity and quality of the follicular reserve with increasing age [2]. Although a decrease in quality has been evidenced by aneuploidy embryos and miscarriage appears to increase with advanced maternal age, it is still necessary to clarify what is referred to by the term follicular quality [3]. The molecular mechanisms that regulate ovarian aging are of great interest, and it has been proposed that the presence and accumulation of senescence cells may contribute to ovarian insufficiency [1].

Senescence promotes tissue remodeling and may contribute to the decline in functions that take place during aging [4]. Lipofuscin is fluorescent material considered the “age pigment” and a marker of senescent cells which increases with age in various tissues, including the ovary [5,6]. The lipids represent an essential component of lipofuscin formation (20–50%) whereas oxidized proteins represent about 30–70% and about 2% of metals [5,7]. Lipofuscin is considered an important source of reactive species oxygen during aging and in the neurogenerative process [5,7]. 

Cellular changes relate to senescence, meaning that the aging process in response to oxidative stress can be detected by nuclear translocation of the nuclear factor-κappa Β (NF-KB) [8]. The maintenance of inactive NF-KB transcription factor complex formed by p50/p52 and p65 (Rel A) in the cytoplasmic cellular compartment is the responsibility of the Ikappa B (IkB) inhibitory protein [9]. Several agents induced by oxidative stress activate the cytoplasmic complex and increase the nuclear translocation and activity of NF-ΚΒ [8,9]. Moreover, NF-ΚΒ activation can be induced by the inhibition of an enzymatic antioxidant system composed of superoxide dismutase (SOD), glutathione peroxidase (GPx), and catalase (CAT) [10]. NF-ΚΒ activation plays an essential role in the activation of various diseases, including cancer of the ovary [11,12].

Coupled with the natural aging process of the ovary, lifestyle-related factors, such as hypercaloric diets, influence ovarian function [13]. The increase in high-calorie diets (high-fat and high-carbohydrate diets) causes metabolic problems and affects ovarian function. It is no surprise that the high-fat diet induces chronic oxidative stress, which contributes to increasing senescent cells and aging [14]. Diverse studies have produced evidence that hypercaloric diets decrease the follicular reserve, promote follicular atresia, and reduce oocyte quality, contributing to fertility problems [15,16]. Table 1 summarizes types of diets and their effects on the follicular reserve level in different animal models. This evidence suggests that hypercaloric diets tend to engender premature aging of the ovary. Thus, further exploration of other aging hallmarks caused by obesogenic diets is imperative. 

The rabbit has proven to be a good model for illustrating altered metabolism induced by obesogenic diets in a way similar to humans [17]. Likewise, it is a good model for high-fat and high-cholesterol diet-induced lipid metabolic disorders [18] and high-fat and sucrose diet-induced adiposity accumulation [19]. In reproductive contexts, the rabbit represents an alternative model for studying early gonadal differentiation [20] and reproductive pathologies [21], and the morphological rearrangement of the cortical region shows changes very similar to those perceived in the aging ovary [22].

**Table 1:** Types of diets and their effects on the follicular reserve.

Diet Type and Experimental Design	Main Findings	Reference
**New Zealand rabbits from 10 weeks of age were fed high-fat, high-cholesterol (HH) diet**	They analyzed follicular populations in the ovary. A higher number of atretic follicles and a reduced number of antral follicles were observed in HH group.	[23]
**Ossabaw mini pigs were fed a high fat/cholesterol/fructose for eight months**	Obese pigs showed more cystic, medium, and ovulatory-size follicles.	[24]
**Sprague Dawley rats were fed a high-fat diet (HFD) for 18 weeks**	Depletion of the ovarian reserve was analyzed. They observed a decrease in primordial follicles whereas the number of growing-to-primordial follicles was higher.	[16]
**C57BL/6J mice strain were fed an HFD for 10 weeks**	Depletion of the ovarian reserve was analyzed. They noticed decreased primordial follicles.	[25]
**C57BL/6J mice strain were fed a HFD for 10 weeks**	They analyzed depletion of the ovarian reserve. Decreased primordial follicles.	[26]
**Mice from the Swiss albino strain were fed an HFD for 9 weeks**	They evaluated the number and developmental morphology of follicles.They observed a reduced number of primordial, while the number of primary, secondary, and tertiary follicles increased with HFD.	[27]
**Wistar rats were fed a high carbohydrate diet (HCD) for 15 days**	A reduced number of primordial, primary, preantral, and antral follicles were observed in the HCD group.	[28]
**Wistar rats were fed a high-sucrose diet (HSD) for 4 months**	The HSD group showed a greater number of atretic antral follicles and cystic follicles. However, the number of follicles between groups did not manifest differences.	[29]

In this study, we used a previously established rabbit model of a high-fat and high-carbohydrate diet (HFCD) [30,31,32] to explore the presence of hallmarks of reminiscent ovarian aging. 

## 2. Materials and Methods

This research was carried out with approval from the local Ethics Committee of the Facultad de Medicina (FM/DI/059/2019) and Instituto de Investigaciones Biomédicas UNAM (ID: 198). The experiments were performed in compliance with the National Institute of Health, Guide for the Care and Use of Laboratory Animals. The rabbits studied in this research were part of a larger study about the effects caused by maternal intake of HFCD. Tissues from the same animals are being used to determine diverse disruptions induced by HFCD, for example, concerning metabolic parameters, body thermogenesis process, circadian rhythmicity, and DNA-induced damage [30,31,32].

We used 10-week-old female rabbits of the European Chinchilla breed (*Oryctolagus cuniculus)*. At this stage, females are at puberty with ovary development showing the growth of antral follicles together with initial formation of the interstitial gland caused by the atresia of these follicles. We used the software Epidat v4.2 to estimate the sampling (www.sergas.es (accessed on 26 June 2019.)); chinchilla rabbits were assigned either to a control group (*n* = 5), fed with a chow standard diet (control group) (Conejo Ganador, Malta Clayton, Mexico) or an experimental group (*n* = 8), fed with a high-fat and carbohydrate diet (HFCD). The HFCD was supplemented with 0.1% cholesterol (Cat. No. C75209, Sigma-Aldrich, St. Louis, MO, USA), 4% soybean oil (Cat. No. S7381, Sigma-Aldrich, St. Louis, MO, USA), and 15% refined sugar (Great Value, Mexico City, Mexico), providing 47% and 10% more energy from fat and carbohydrates. Animals in the control group received 3.8% and 47.8% of kcal from fat and carbohydrates than standard diet (Table 2) [31]. All female rabbits were fed until 25 weeks of age when they reach sexual maturity. Animals were sacrificed by means of an intravenous pentobarbital overdose (60 mg/Kg) (Pisa Agropecuaria, Nuevo México, Mexico) applied to the marginal ear vein. The left ovary was removed and cut in half along a transverse plane and fixed in 4% paraformaldehyde (Cat. No. 158127, Sigma-Aldrich, St. Louis, MO, USA) overnight. Samples were embedded in paraplast plus (Cat. No. P3683, Sigma-Aldrich, St. Louis, MO, USA). Sections were used for an ovarian follicle count, lipofuscin detection, and either immunohistochemistry or immunofluorescence. 

### 2.1. Masson’s Trichrome Stain and Ovarian Follicle Counting

A rotary microtome was used to section tissues at 7 µm, and alternate slices were mounted on glass slides for histology, Sudan Black B (SBB), immunohistochemistry, and immunofluorescence. Histological analysis was carried out using Masson’s trichrome stain [33]. Photo stitching was undertaken manually, using images collected with a Nikon microscope model Eclipse Ni-U (Nikon, Tokyo, Japan), uniting 13 to 15 images. The stained sections were used for determining and quantifying ovarian follicles in both groups. Ovarian follicles were assigned to a stage of folliculogenesis: Primordial follicle: oocyte with a single layer of squamous granulosa cells; Transition follicle: oocyte with a layer of both squamous and cuboidal granulosa cells; Primary follicle: oocyte surrounded by a layer of cuboidal granulosa cells; Preantral follicle: oocyte with two or more layers of granulosa cells; early and late antral follicle; atretic follicles; corpus luteum; and remnants of atretic follicles. The count was undertaken in eight fields per section at 10× magnification with five sections per ovary. The counts from each section were added together to obtain the number of follicles per ovary.

### 2.2. Lipofuscin Detection and Analysis

Lipofuscin detection was performed with SBB staining. Briefly, paraffin was removed and rehydrated in a descending ethanol series. Slides were stained with SBB 0.7% (Cat. No. 199664, Sigma-Aldrich, St. Louis, MO, USA) in 70% ethanol for 2 min. Afterward, the sections were briefly rinsed in 50% ethanol and distilled water. Finally, they were mounted using an aqueous mounting medium (Cat. No. F4680, Sigma-Aldrich, St. Louis, MO, USA). The sections were collected using a Nikon microscope model Eclipse Ni-U (Nikon, Japan) attached to a digital camera at 10× magnification using NIS-Elements software. We analyzed the qualitative accumulation of lipofuscin staining with SBB in several cell types for all samples: epithelium, stroma, granulosa cells of different follicle types, oocyte, and interstitial gland. Three arbitrary categories were established: [+] low stain, [++] moderate stain, and [+++] high stain [34]. Next, each cell type was assigned to one of these three categories. In addition to qualitative analyses, lipofuscin quantification in images of interstitial gland was performed using ImageJ software. Images were converted to grayscale, the scale was set, and using the threshold option, lipofuscin content was highlighted. Then, a previously established rectangular area (5581.14 µm^2^) was randomly positioned in the interstitial gland region of each image. We analyzed five sections per ovary (nine areas per section).

### 2.3. Immunohistochemistry and Immunofluorescence 

We prehydrated paraplast sections and performed heat-induced antigen unmasking in the decloaker chamber for 15 min at 110 °C in 1X Diva (Cat. No. DV2004, Biocare Medical, New York City, NY, USA). Subsequently, the slides were incubated with a blocking solution (10% horse serum in 0.5% triton/TBS (Cat. No. 97064-338NP, VWR Life Science, Missouri, TX, USA) for 2 h. Endogenous peroxidases and biotin were quenched with Bloxall (Cat. No. SP-6000, Vector Labs, Newark, CA, USA)) for 20 min and Streptavidin/Biotin Blocking Kit (Cat. No. SP2002, Vector Labs, Newark, CA, USA) for 15 min, respectively. These were then incubated with primary antibodies (see Table 3) at 4 °C, overnight. Negative controls were incubated without primary antibodies. Tissue sections were washed with TBS three times for 5 min. The resulting secondary antibodies were incubated for 20 min at room temperature. Slides were incubated with ImmPACT™ DAB Peroxidase Substrate Kit (Cat. No. SK-4105, Vector Labs, Newark, CA, USA) to reveal the color of enzyme-substrate antibody staining. Subsequently, these were treated with acetate buffer for 10 min and counterstained with hematoxylin for 1 min. Slides were dehydrated, transferred to xylene for 5 min, and mounted with Sub-X Mounting Medium (Cat. No. 3801741, Leica Bio, Richmond, IL, USA). The sections were documented using a Nikon microscope model Eclipse Ni-U (Nikon, Japan) attached to a digital camera at 10x magnification using NIS-Elements software. For quantification, optical density (OD) was determined for each sample using Fiji software as described previously; immunostaining was measured as mean gray value in the interstitial gland region. We established an area of 5384.67 µm^2^ (10×) that was randomly positioned in interstitial gland zone and 100,000 µm^2^ (40×) in surface epithelium from each image. Optical density was calculated using the formula OD = log (max intensity/mean intensity) where max intensity is 255 for 8-bit images.

We performed immunofluorescence to observe cellular localization of NF-ΚΒ. After dewaxing, the slides were washed in TBS. Heat-induced antigen retrieval was performed by placing the slides in a decloaker chamber (Cat. No. DV2004, Biocare Medical, CA, USA) in 1X Diva Decloaker (Biocare Medical, CA, USA) 10 for 15 min at 110 °C. Sections were incubated with 10% horse serum for two hours to block nonspecific binding to proteins. Slices were incubated with primary antibody NF-ΚΒ (see Table 2) overnight at 4 °C. Negative controls were incubated without primary antibodies. Subsequently, slides were washed with TBS and secondary antibody was incubated for 20 min at room temperature. Cell nuclei were counterstained DAPI (5 µg/ µL) for 10 min. To quench autofluorescence, sections were incubated with 1X TrueBlack Lipofuscin (Cat. No. 23007, Biotium, Inc., Fremont, CA, USA). Finally, tissue sections were mounted using Dako Fluorescence Mounting Medium (Cat. No. S302380, Dako, Santa Clara, CA, USA). The slides were mounted with an aqueous mounting medium. Sections were observed and captured under LSM 880 Zeiss confocal microscope for display purposes and merged multichannel images were constructed: NF-ΚΒ (green) and DAPI-stained nuclei cells (blue). The nuclear localization of NF-ΚΒ (40×) was determined by mean fluorescence intensity in the nucleus of cells of surface epithelium. Using Fiji software, we created a binary mask of the nuclei of surface epithelium in channel DAPI to establish the region of interest (ROI) in the NF-ΚΒ channel.

### 2.4. Statistical Analysis

Statistical differences between groups were established, applying an unpaired Student’s *t*-test using Instat Software Vers 3.05. Data are represented as mean ± standard error of the mean (SEM). Statistical significance was set at *p* < 0.05 bimarginally.

## 3. Results

### 3.1. HFCD Caused a Decreased in the Ovary’s Follicular Reserve

**Figure 1:**
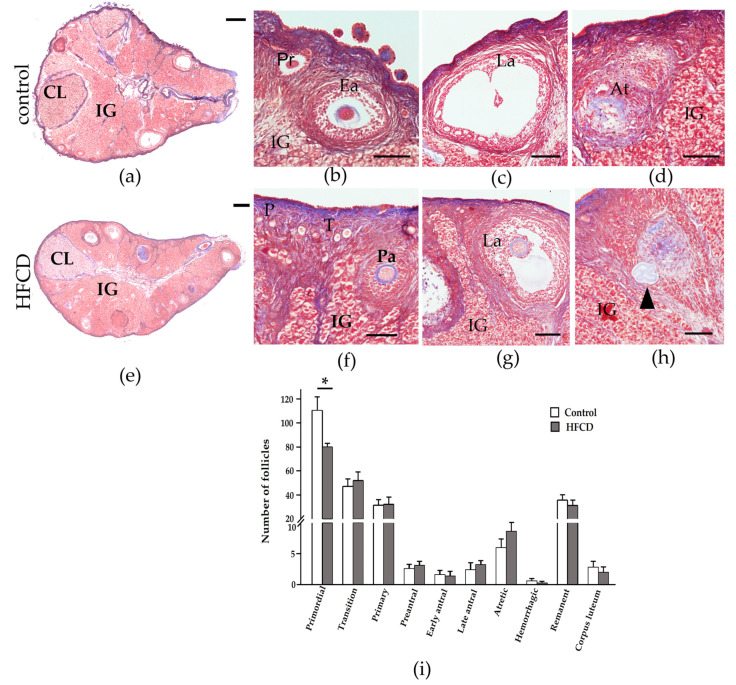
Representative photo stitching of Masson’s trichrome staining ovary sections and follicular counting of rabbits fed either a control diet or HFCD. (**a**,**e**) Ovarian histology. (**b**–**d**,**f**–**h**) Amplification showing primordial (P), Transition (T), Primary (Pr), Preantral (Pa), Early antral (Ea), Late antral (La), atretic follicle remnants (black arrow) and Atretic follicles (At), corpus luteum (CL), and Interstitial gland (IG). Scale: (**a**,**e**): 500 µm. (**b**–**d**,**f**–**h**): 100 µm. (**i**) Histogram of the mean number of follicles depending on the type of rabbit ovarian follicle, fed either a control diet or HFCD. Each box represents the mean value ± ESM for each group. Statistical significance was set at * *p* < 0.05.

We evaluated follicular populations in the control ovary and that of the HFCD rabbits. Figure 1 shows a representative photo stitching of Masson’s trichrome staining of ovary sections, including from rabbits fed a control diet and those fed a HFCD, in order to evaluate follicular populations in both these contexts. The number of primordial follicles showed a significant decrease in the HFCD group compared to the control group (* *p* < 0.05). However, the growing follicles did not show significant differences (Figure 1i).

### 3.2. HFCD Increased the Presence of Lipofuscin in the Interstitial Gland

In order to determine the presence of aging markers, we used SBB staining to identify lipofuscin (age pigment) accumulation in the control and HFCD ovaries. Lipofuscin was detected in several types of cells in the ovaries of both the control and HFCD (Table 4). A greater increase in lipofuscin was evident in the interstitial gland whereas the stroma, surface epithelium primordial, and primary follicle showed less accumulation. Qualitative analyses showed higher lipofuscin accumulation in the interstitial gland of the HFCD group than in the control group (Table 4).

**Figure 2:**
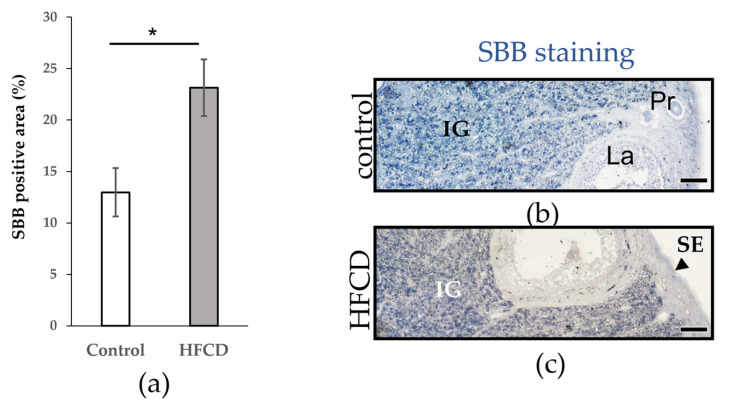
Semiquantification of lipofuscin in the ovarian interstitial gland. (**a**) Semiquantification of lipofuscin was measured as a percentage of the area stained with 0.7% SBB in the ovarian interstitial gland of chinchilla rabbit fed with either a control diet (n = 5) or HFCD (n = 8) * *p* < 0.05. Vertical bars indicate the standard error of the mean. (**b**,**c**) Representative SBB staining sections related to the control diet or HFCD. Scale bar: 50 µm. Primary follicle (Pr), Late antral (La), surface epithelium (SE, black arrow), and interstitial gland (IG).

We used SBB staining to corroborate the increase in the interstitial gland of HFCD ovaries. Analysis revealed conformity between methods, indicating and confirming the increased significance of lipofuscin accumulation in the interstitial gland of HFCD in contrast to control ovaries (* *p* < 0.05) (Figure 2). 

**Figure 3:**
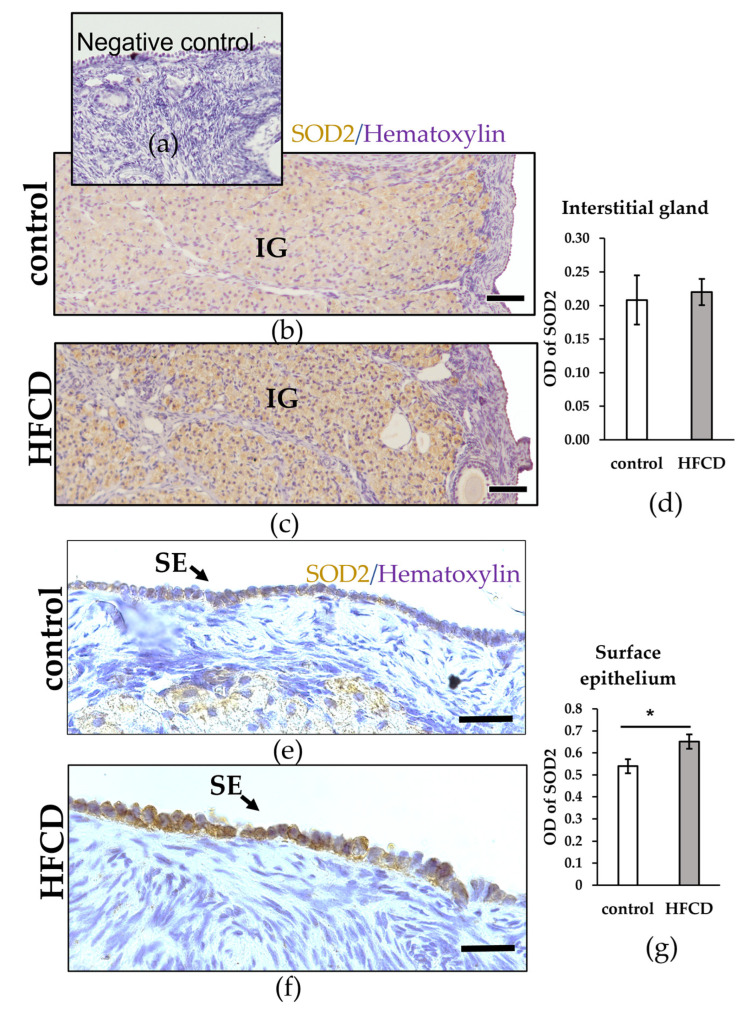
SOD 2 protein localization and levels in ovaries. (**a**) Negative control without primary antibody; no signal detected. Immunochemical localization (brown) in the ovarian interstitial gland (**b**,**c**) and surface epithelium (**e**,**f**) of chinchilla rabbits, fed with either a control diet or with HFCD. The nuclei are stained with hematoxylin (blue). Scale bars: (**b**,**c**), 100 µm; (**e**,**f**), 25 µm. (**d**,**g**) Densitometric quantification performed on immunohistochemical localization of SOD 2 of ovarian sections of chinchilla rabbit fed with either a control diet or with HFCD. Data are expressed as mean ± ESM * *p* < 0.05.

Due to great steroidogenic activity, the interstitial gland has been proposed as a source of oxidative stress, so we decided to explore the impact of an HFCD on the antioxidant barrier. Observing the expression of SOD 2, no significant differences were detected in the interstitial gland. However, the surface epithelium cells were higher expressed of the HFCD in contrast to control ovaries (* *p* < 0.05) (Figure 3).

### 3.3. HFCD Induces NF-ΚΒ p65 Nuclear Translocation in Cells of Surface Epithelium

**Figure 4:**
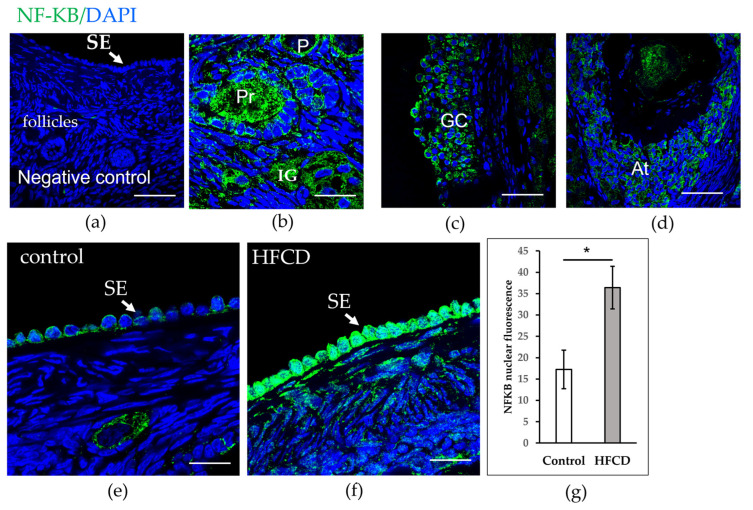
NF-ΚΒ protein localization and levels in ovaries of chinchilla rabbit. (**a**) Negative control without primary antibody, no signal detected. (**b**–**d**) Cytoplasmic localization of NF-ΚΒ (green) in interstitial gland (IG), Primordial follicles (P), Primary (Pr), mural granulosa cells (GC), and Atretic follicle (At). The nuclei are stained with DAPI (blue). (**e**) Cytoplasmic localization of NF-ΚΒ in surface epithelium (SE) and oocyte of the primordial follicle of chinchilla rabbit, fed with a control diet, and (**f**) cytoplasmic and nuclear localization of NF-ΚΒ (green) in the ovarian surface epithelium of chinchilla rabbit fed with HFCD. Scale bars: (**a**–**d**) 50 µm; (**e**,**f**) 100 µm. (**g**) Densitometric quantification was performed on nuclear NF-ΚΒ expression of the surface epithelium of chinchilla rabbit fed with either a control diet or HFCD. Data are expressed as mean ± ESM * *p* < 0.05.

SOD 2 is the most famous NF-KB target. We thus explored the impact of HFCD on the immunolocalization of NF-ΚΒ using confocal microscopy. Figure 4 shows cytoplasmic localization of NF-ΚΒ in the surface epithelium, oocyte of the primordial follicle of chinchilla rabbit fed with a control diet, and cytoplasmic granulosa cells. However, only the nuclei of ovarian surface epithelium cells of chinchilla rabbits fed with the HFCD manifested strong immunoreactivity nuclear localization of NF-ΚΒ. We quantified nuclear NF-KB staining, which revealed a significant increase in nuclear fluorescence in the cells of the surface epithelium for the HFCD group that was not evident among rabbits fed the control diet (Figure 4).

## 4. Discussion

In addition to infertility, the aging ovary is associated with several age-related diseases, including cardiovascular disease, osteoporosis, hypertension, and ovarian cancer. It is thus crucial to understand the mechanisms that promote its deterioration [35]. Lipofuscin is evident in the ovaries of mice of advanced age. However, it was not detected in the oviduct or uterine horns, corroborating that the ovary is the first reproductive organ to undergo aging [36].

The adiposity induced by hypercaloric diets can accelerate age-related ovarian disruption. Similarly, there is increasing evidence that obesity reduces ovarian reserve in young animals [16,23,24,25,26,27,28,29]. In this sense, this research has shown that an HFCD induced a decrease in follicular reserve, similar to that observed in other animal models. 

We also attempted to determine whether an HFCD promotes the accumulation of senescent cells. During senescence, many proteins, contents of old lysosomes or lysosomal biogenesis and residual bodies, known as lipofuscins, are accumulated. Lipofuscin is considered a marker of senescent cells in various tissues [4]. Moreover, hypercaloric diets may exacerbate senescent cell-type aging [35,37]. Recently, Hense and colleagues reported that obese mice showed a more significant presence of senescent cells, characterized by lipofuscin accumulation and elevated p21 and p16 expression (cell cycle inhibitors) [35]. However, the authors failed to determine the type of ovarian cells that become senescent. In this study, we found that the ovarian interstitial gland of rabbits fed with an HFCD manifested a more significant accumulation of lipofuscin. To our knowledge, this is the first report demonstrating that the ovary’s senescent cells are formed in the interstitial gland. We previously reported that the hypertrophic interstitial gland is linked to the aging ovary [22]. During the period when the ovary is aging, the interstitial gland occupies the cortical region while the follicular reserve decays [22].

Xu and colleagues demonstrated that transplanting fewer senescent cells into young mice induced reduced health and life span, an effect which can be contrasted with senolytic treatment [37]. In this sense, Hence and colleagues evaluated the impact of senolytic treatment on ovarian senescence induced by obesity and discovered reduced effects. In 1902, for the first time, Limon described the formation of the interstitial gland, derived from the theca cells of atretic follicles of the human ovary [38]. Considering that the development of the interstitial gland in rabbits is very similar to that described for the human ovary [22], it is possible to postulate that the accumulation of senescent cells promoted by hypercaloric diets accumulates in the interstitial gland in the human ovary. Therefore, performing the surgical removal of this tissue may be possible with a considerable probability of reversing harmful effects. 

Due to its high steroidogenic activity, the interstitial gland is associated with oxidative stress. Moreover, lipofuscin is considered an essential source of age-related oxidants [39]. Oxidative stress may modulate NF-ΚΒ response to promote survival; one of the most important mechanisms of NF-ΚΒ is to increase the expression of inducible manganese superoxide dismutase (SOD 2). SOD 2 is a mitochondrial enzyme and one of the most important elements in the antioxidant barrier, converting O_2_ into H_2_O_2_ [39]. Our objective was thus to determine changes in the expression and localization of SOD 2 and NF-KB (p65). Surprisingly, HFCD did not induce changes in the expression of SOD 2 in the interstitial gland; however, greater expression of surface epithelium cells resulted. The ovarian surface epithelium comprises a single layer of flat or cuboidal cells. Research interest concerning the surface epithelium has been generated by its susceptibility to malignancy. Approximately 90% of human ovarian cancer arises in the ovarian surface epithelium [40]. In all body tissue, the normal function requires a balance between reactive oxygen species and antioxidants. Upsetting this balance alters the function of systems, including reproductive function [41]. In addition to ovulation [42], the interstitial gland may contribute to inflammatory mediators and reactive oxygen species. Likewise, an HFCD causes an increase in the expression of NF-KB in the nucleus of the ovarian surface epithelium cells. Regulation of NF-ΚΒ is mediated by reactive oxygen species (ROS) production, with RelA (p65) manifesting the most sensitive reaction to activation by ROS.

This study observed increased nuclear expression of NF-KB (p65) in the ovarian surface epithelium. The surface epithelium is a mitotically active tissue that repairs itself following each ovulation [40]. Mitosis is the mechanism that cells use to prevent the accumulation of lipofuscin [43]. However, the increased presence of senescent cells in the ovarian interstitial gland may promote the upregulation and translocation of NF-KB (relA), resulting in increased SOD 2 expression. Greater expression of SOD 2 induced by increased nuclear translocation of NF-ΚΒ may be a response on the part of the surface epithelium, as it attempts to provide protection from oxidative stress, induced by the interstitial gland. Recently Gao and colleagues showed p-NF-KB expression and proinflammatory cytokine in the reproductive organs of obese female mice [44]. Moreover, the NF-ΚΒ pathway is associated with ovarian cancer [11,12]. Activating the canonical pathway of NF-ΚΒ regulates downstream transcriptional targets of potent antioxidants, such as SOD 2. This pathway is activated principally by TNF and interleukins [11,12]. 

## 5. Conclusions

We conclude that an HFCD induces a greater accumulation of senescent cells in the interstitial gland, promoting the appearance of features reminiscent of ovarian aging. However, the activation mechanism of NF-KB caused by an HFCD, which may be stress-responsive and generated by the interstitial gland, requires further study. We consider that the rabbit is a model with great potential for identifying and characterizing the possible secretory phenotype of the senescent cells in the ovarian interstitial gland induced by aging and hypercaloric diets. 

## Figures and Tables

**Table 2:** Nutrient composition of the control diet and HFCD [30].

Composition	Control Diet (%)	HFCD (%)
Digestive carbohydrates (Nitrogen-free extract)	47.8	52.6
Fat (ether extract)	3.8	5.6
Crude protein	15.7	11.2
Crude fiber	15.3	10.2
Mineral (ashes)	11.5	7.6
Humidity	5.6	12.8

**Table 3:** Antibodies used for immunolocalization.

Primary Antibodies	Host	Dilution	Cat. Number	Supplier
Superoxide dismutase 2 (SOD 2)	Mouse	1:50	GTX630558	GeneTex
NF-ΚΒ p65 (G-8)	Mouse	1:60	sc-398442	Santa Cruz Biotechnology
Secondary antibodies				
Biotinylated anti-Mouse IgG	---	1:500	BMK-2202	Vector Laboratories
Alexa Fluor 488 anti-mouse IgG	Donkey	1:500	A21202	Thermo Fisher Scientific
HRP-conjugated streptavidin	---	1:500	SA-5004	Vector Laboratories

**Table 4:** Qualitative determination of lipofuscin stained SBB in different cell types in ovaries of both the control and HFCD.

	Control	HFCD
Stroma	+	+
Surface epithelium	+	+
Primordial follicles	+	+
Primary follicles	+	+
Secondary follicles (granulosa cells)	++	++
Late secondary follicles (granulosa cells)	++	++
Oocytes	++	++
Cumulus cells	+++	+++
Interstitial gland	++	+++

Arbitrary categories: [+] low stain, [++] moderate stain, and [+++] high stain.

## Data Availability

Not applicable.
